# Breast MRI in patients after breast conserving surgery with sentinel node procedure using a superparamagnetic tracer

**DOI:** 10.1186/s41747-021-00257-7

**Published:** 2022-01-27

**Authors:** Anke Christenhusz, Joost J. Pouw, Frank F. J. Simonis, Michael Douek, Muneer Ahmed, Joost M. Klaase, Anneriet E. Dassen, Caroline A. H. Klazen, Margreet C. van der Schaaf, Bernard ten Haken, Lejla Alic

**Affiliations:** 1 Magnetic Detection & Imaging group, Technical Medical Centre, University of Twente, Enschede, The Netherlands; 2 Department of Surgery Medisch Spectrum Twente, Enschede, The Netherlands; 3 Nuffield Department of Surgical Sciences, University of Oxford, Oxford, UK; 4 Division of Surgery and Interventional Science, University College London, Royal Free Hospital, London, UK; 5 Department of Radiology, Medisch Spectrum Twente, Enschede, The Netherlands

**Keywords:** Artefacts, Breast cancer, Magnetic resonance imaging, Superparamagnetic iron oxide nanoparticles, Sentinel lymph node biopsy

## Abstract

**Background:**

A procedure for sentinel lymph node biopsy (SLNB) using superparamagnetic iron-oxide (SPIO) nanoparticles and intraoperative sentinel lymph node (SLN) detection was developed to overcome drawbacks associated with the current standard-of-care SLNB. However, residual SPIO nanoparticles can result in void artefacts at follow-up magnetic resonance imaging (MRI) scans. We present a grading protocol to quantitatively assess the severity of these artefacts and offer an option to minimise the impact of SPIO nanoparticles on diagnostic imaging.

**Methods:**

Follow-up mammography and MRI of two patient groups after a magnetic SLNB were included in the study. They received a 2-mL subareolar dose of SPIO (high-dose, HD) or a 0.1-mL intratumoural dose of SPIO (low-dose, LD). Follow-up mammography and MRI after magnetic SLNB were acquired within 4 years after breast conserving surgery (BCS). Two radiologists with over 10-year experience in breast imaging assessed the images and analysed the void artefacts and their impact on diagnostic follow-up.

**Results:**

A total of 19 patients were included (HD, *n* = 13; LD, *n* = 6). In the HD group, 9/13 patients displayed an artefact on T1-weighted images up to 3.6 years after the procedure, while no impact of the SPIO remnants was observed in the LD group.

**Conclusions:**

SLNB using a 2-mL subareolar dose of magnetic tracer in patients undergoing BCS resulted in residual artefacts in the breast in the majority of patients, which may hamper follow-up MRI. This can be avoided by using a 0.1-mL intratumoural dose.

## Key points

- The residual susceptibility artefacts after a high dose of subareolar superparamagnetic iron-oxide (SPIO) injection hinder the diagnostic value of follow-up magnetic resonance imaging (MRI).
- The residual susceptibility artefacts on follow-up MRI are preventable by using a low-dose intratumoural SPIO injection.

## Background

In the Netherlands, almost 15,000 people receive the diagnosis of breast cancer annually, and one out of five will develop metastases [1]. For a good treatment and prognosis, early detection of metastases is important. The sentinel lymph node biopsy (SLNB) was developed as a minimally invasive procedure for lymph node staging to avoid axillary dissection in breast cancer patients [2]. SLNB using a radioisotope tracer (^99m^Tc-nanocolloid) to identify the SLN, or combined with a blue dye, is the worldwide standard-of-care for axillary staging of patients with early-stage breast cancer.

A new technique based on magnetism was developed for intraoperative SLN detection to avoid radiation-associated issues, such as radiation exposure, supply of the radioisotopes, legislative requirements, and logistical challenges [3]. The SentiMAG® magnetometer, in combination with a superparamagnetic iron-oxide (SPIO) tracer (Sienna+®/Magtrace, 27 mg iron per mL; Endomagnetics, Cambridge, UK), was evaluated for SLNB in several clinical trials. They have shown the non-inferiority of magnetic detection compared to the radioactive standard [4–11]. Furthermore, several clinical trials have investigated different injection sites, such as subareolar [4], intratumoural [12], and peritumoural [13], and using different doses of magnetic tracer, such as 2 mL [4] and 1.5 mL [14].

Currently, breast MRI is not part of the standard care for postoperative follow-up after breast conserving surgery (BCS) [15, 16]. However, it may be valuable to assess suspected recurrence when mammography and/or ultrasound bring no clarity or produce non-corroborative findings. In such patients, those with extremely dense breast tissue or with genetic alterations (such as those regarding *BRCA*1 or *BRCA2* genes), MRI may contribute to the management of the disease [17, 18]. However, in cases where the residual magnetic tracer is retained after BCS, its presence can lead to artefacts in the follow-up MRI examinations [19, 20]. Currently, the manufacturer of the magnetic tracer also warns about the long-term artefacts in MRI studies following the magnetic procedure for SLNB [21].

Even though it is currently well-known that magnetic tracers may introduce MRI artefacts, it is not known how long these artefacts persist or whether they can be avoided using a different injection site and dose of the tracer. Huizing et al. [22] retrospectively identified injection site void artefacts in 10 postoperative dynamic contrast-enhanced breast MRIs of participants in a magnetic SLNB trial. However, there is no standard grading protocol for susceptibility artefacts and their impact on diagnostic imaging. A previous publication [23] qualitatively evaluated the void artefacts and their impact on diagnostics. Current research is a quantitative evaluation of the void artefacts due to the magnetic SLNB procedure based on injection site and dose, and evaluation of how such artefacts can be prevented in the future.

For this purpose, the follow-up MRI data from trial participants in two separate multicentre studies with different doses of SPIO (27 mg iron per mL) were compared:

- SentiMAG multicentre trial [4]: 2-mL subareolar SPIO injection to compare SLNB using magnetic nanoparticles and current standard-of-care tracers, referred to as the high dose (HD) group;
- MagSNOLL multicentre trial [12]: 0.1-mL intratumoural SPIO injection for a magnetic procedure for SLNB and occult lesion localisation, referred to as the low dose (LD) group.

We hypothesised that an intratumoural LD leads to a decrease in the residual magnetic tracer after surgery, eliminating artefacts in the follow-up MRI examinations, while still allowing localisation of the SLN and non-palpable tumours using magnetic detection. Intratumoural or peritumoural injection for the purpose of decreasing the residual magnetic tracer is in the meantime common practice in the field [14]. All patients treated with BCS and the magnetically guided SLNB at our centre were invited for a postoperative MRI to evaluate the influence of injection site and dosage.

The purpose of this prospective study was to determine if the tracer used in the magnetic SLNBs results in long-lasting MRI void artefacts and whether these artefacts could be avoided by using a LD intratumoural injection. Additionally, we also developed a clinical grading protocol to assess the severity of these artefacts.

## Methods

### Patients

All patients referred for breast surgery with axillary lymph node staging, received the current standard-of-care SLNB using a radioactive tracer ^99m^Tc and patent blue dye. In the period between May 2012 and March 2015, 76 patients were included in the HD group (SentiMAG trial | NL39018.044.11) [4] and low dose group (MagSNOLL trial | NL49350.044.14) [12] in which the magnetic SPIO tracer Sienna+® (27 mg iron per mL; Endomagnetics, Cambridge, UK) was injected into the breast. In the HD group, patients received a subareolar 2-mL injection of SPIO nanoparticles diluted in 3-mL saline, and a treatment for perioperative wire-guided localisation of the primary tumour. In the LD group, the patients were injected intratumourally with 0.1 mL of SPIO nanoparticles that were utilised for perioperative tumour detection as well as magnetic SLNB. None of the patients scheduled for SLNB had signs of local metastasis in the SLN based on both clinical and radiological evaluation.

Reasons for exclusion from both studies were pregnancy, known intolerance or hypersensitivity to iron or dextran compounds, contraindications for MRI, and implants on the chest wall. The size of the excised specimen, after BCS with SLNB, was assessed by a pathologist in three spatial dimensions. All patients included in this study provided written informed consent, allowing the acquisition and analysis of MRI data after BCS under the approval of a local ethics committee.

### Diagnostic imaging

A two-view mammography was obtained using Selenia Dimensions 3D (Hologic, Bedford, MA, USA) in two directions: craniocaudal view and mediolateral oblique view. Preoperative mammography was used to assess the tumour size. Postoperative mammography was acquired during routine, annual follow-up and was additionally assessed for the presence of long-term SPIO artefacts.

MRI data was acquired with a 1.5-T MRI scanner (Intera, Philips Medical Systems, Best, the Netherlands) using a dedicated breast coil. T1-weighted turbo spin-echo sequences were acquired in the axial and coronal planes, while T2^*^-weighted gradient echo sequences were acquired in the axial plane. Table 1 summarises the acquisition parameters. All diagnostic images (mammography and MRI) were analysed using the workstation (DynaCAD®, Philips Healthcare, Best, the Netherlands).

**Table 1:** Technical parameters of magnetic resonance sequences

	Axial T1-weighted turbo spin-echo	Coronal T1-weighted turbo spin-echo	Axial T2*-weighted fast field-echo
Repetition time (ms)	734	727	500
Echo time (ms)	16	16	4.6
Flip angle (°)	90	90	18
Slice thickness (mm)	3	3	3
Bandwidth (Hz/pixel)	316	316	154
Field of view (mm^2^)	300 × 300	300 × 300	298 × 298
Voxel size (mm^2^)	0.94 × 0.94	0.94 × 0.94	0.75 × 0.75

### Data analysis

Considering there is no standardised protocol to assess the extent of void artefacts and its impact on diagnostic follow-up MRI, the following grading scale for the assessment of void artefacts after magnetic SLNB was developed. Two radiologists with over 10 years of experience in breast imaging (M.S. and C.K.) assessed the fibroglandular tissue (FGT), void artefacts and axillary nodes. Both readers were blinded to the injected dose groups.

**Fig. 1:**
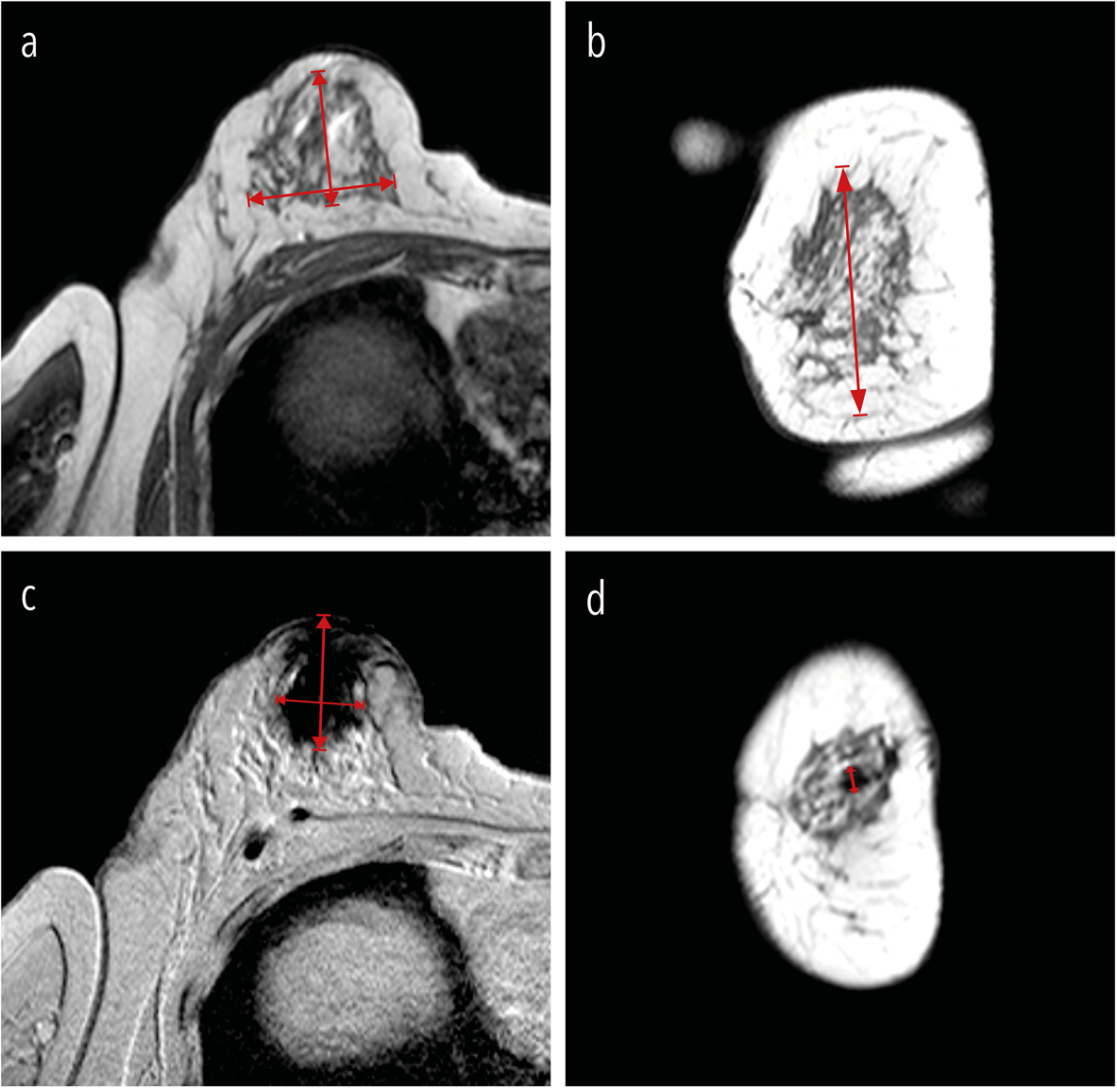
FGT and artefact measurements. The overall volume of FGT was manually assessed by both radiologists independently on T1-weighted images in three orthogonal planes. Dg_1_ and Dg_2_ were the maximal extension measured in the axial plane (**a**). Dg_3_ was the maximal extension measured in the coronal plane (**b**). The artefact diameter assessed on both T1- and T2*-weighted images in three orthogonal planes are shown in **c** (Da_1_, Da_2_) and **d** (Da_3_)

The artefact diameter on both T1- and T2*-weighted images in three orthogonal planes (Da_1_, Da_2_, and Da_3_) was manually assessed by both radiologists independently. The overall volume of FGT in three orthogonal planes (Dg_1_, Dg_2_, and Dg_3_) was manually assessed by both radiologists independently on T1-weighted images (Fig. 1). The volumes of artefact and FGT were subsequently estimated assuming an ellipsoid form. Interobserver variability in assessing volumes was evaluated as absolute and relative measure [24]. The qualitative grading by a 4-point scale in consensus on axial T1- and T2*-weighted scans was assessed according the following scale:

- 0 (no artefact), in the case artefacts smaller or equal to 5 mm in diameter assessed on either T1- and T2*-weighted scans;
- 1 (good diagnostic quality), in the case of artefacts visible only in the superficial subcutaneous tissue (assessed on either T1- and T2*-weighted scans), or artefacts only observable on T2*-weighted images;
- 2 (impaired but still readable), in the case of artefacts observable on T1-weighted images with a volumetric size ≤ 30% of the overall volume of FGT;
- 3 (hampered clinical assessment), in the case of artefacts observable on T1-weighted images with a volumetric size > 30% of the overall volume of FGT.

The artefact location was qualitatively assesses as 0 (outside FGT) or 1 (inside FGT). The SPIO residue in axillary lymph nodes was qualitatively assessed as 0 (no SPIO residue in axillary lymph nodes visible) or 1 (SPIO residue in axillary lymph nodes).

### Statistical analysis

To exclude the possibility of results originating from the differences between the HD and LD group in primary tumour size as assessed at preoperative mammography or volume of the excised pathology specimen, these differences were assessed using Student *t*-test after testing assumption of normality by Shapiro-Wilk normality test. Fisher exact test was used to compare the difference between the HD and LD group in terms of the presence of void artefacts. Qualitative grading 0, 1, and 2 was merged as not hampering clinical assessment for the purposes of this test. The Mann–Whitney *U* test or Student-*t* test (verified by Shapiro-Wilk test) was used to assess the interobserver differences between the HD and LD groups when evaluating the volume of FGT or artefact. Eta-squared, a measure of effect size for use in analysis of variance, was used as a descriptive measure to assess the strength of association between the severity of artefacts and the time from injection.

## Results

### Patient and tumour characteristics

**Fig. 2:**
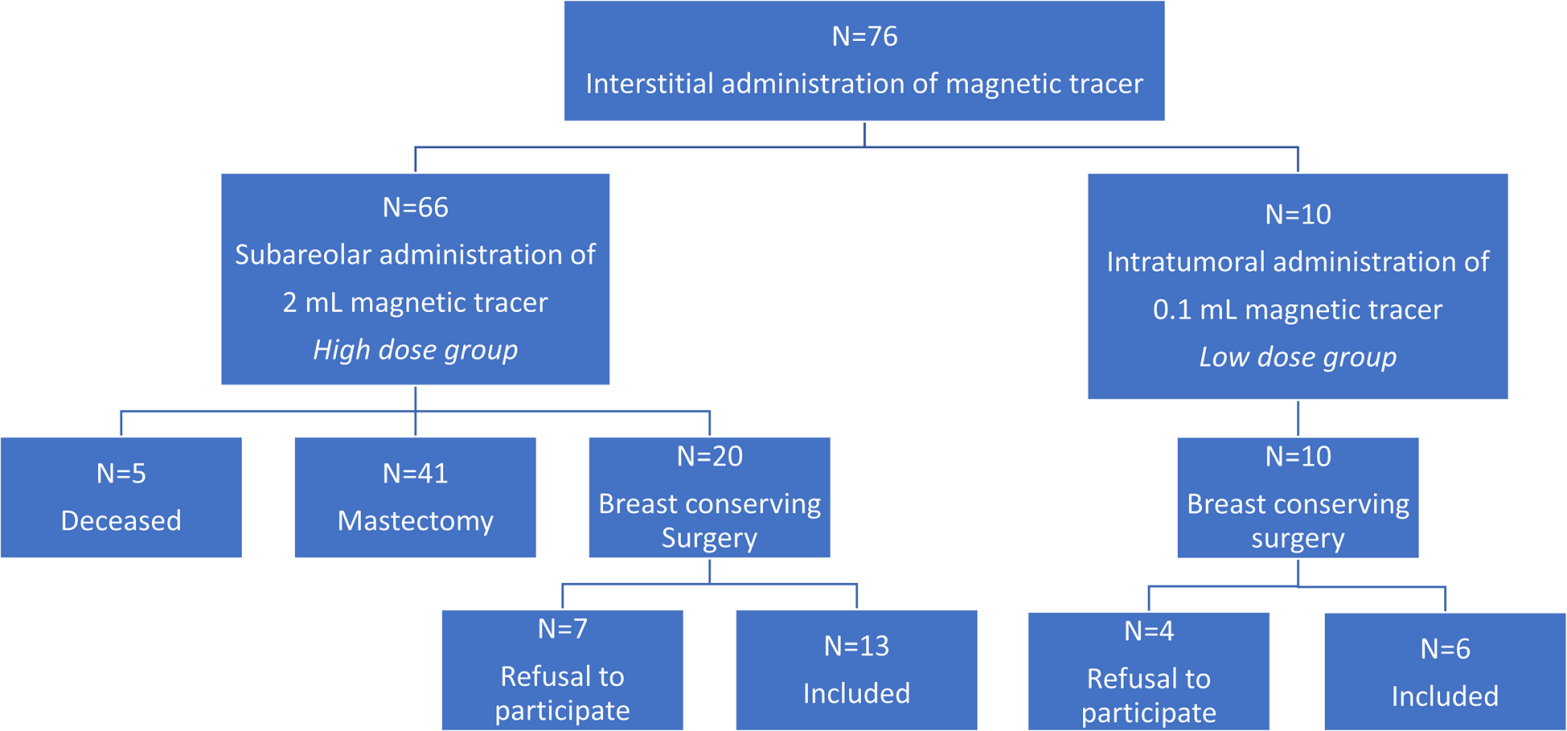
Flow chart of the included and excluded patients

Of the 76 patients invited for a follow-up MRI, 41 patients had had a mastectomy, five were lost to follow-up due to death, and 11 patients refused to participate. Consequently, 19 patients with BCS including magnetic SLNB were included in the follow-up breast MRI study (Fig. 2): 13 participants in the HD group and six participants in the LD group. The follow-up MRI after SLNB was acquired within 4 years in the HD group and within 1 year in the LD group. The study population is presented in Fig. 2 and summarised in Table 2.

**Table 2:** Patient and tumour characteristics

Variable	HD group (*n* = 13)	LD group (*n* = 6)
Age (years)		
45–50	2	0
51–69	9	5
≥ 70	2	1
Tumour stage		
T1	12	4
T2	1	1
T3	0	0
Tis (ductal carcinoma in situ)	0	1
Tumour type		
Invasive, no special type	12	5
Invasive, mucinous	1	0
Ductal carcinoma in situ	0	1

In the HD group, nine patients received the SPIO injection on the day of surgery, and four patients received the SPIO injection 1 day before surgery. In the LD group, all patients received the injection one day before surgery. All 19 SLNB procedures were performed successfully with no differences in terms of detection rate during surgery between the two groups. There was no significant difference in tumour size on the preoperative mammography or the size of the excised specimen between the two patient groups (Table 3).

**Table 3:** Characteristics

	HD group Mean (range)	LD group Mean (range)
Age at surgery (years)	58.5 (46–76)	64.3 (58–71)
Time surgery to follow-up MRI (years)	2.9 (2.5–3.4)	0.6 (0.5–0.7)
Tumour size (mm)^a^	14.5 (7–25)	10.5 (4–21)
Lump volume (cm^3^)^b^	52.3 (22.1–82.9)	47 (31.6–72.9)

Data are presented as mean and range

^a^Assessed with preoperative mammography

^b^Assessed at pathology specimen in three spatial dimensions

During clinical follow-up, two patients (one patient from the HD and one patient from the LD group) had been diagnosed with contralateral breast cancer, with one patient from the LD group dying 5 years after surgery due to metastatic lung cancer. In another patient from the LD group, with invasive breast cancer (no special type) and ductal carcinoma in situ, recurrence and local metastases were detected during follow-up 4 years after surgery.

### Qualitative grading

The intensity changes, which are due to the remaining iron accumulation, were less prominent on T1-weighted images than on T2*-weighted images. For T2*-weighted images, especially those with grade 3 artefacts, large areas of hypointense signal severely hampered or inhibited the MRI diagnostic follow-up. The shape of these artefacts was is highly irregular, making it also difficult to assess the artefact dimensions.

In the cases of grade 2 artefact, T1-weighted images showed a morphological change with a hypointense signal, while T2*-weighted images showed a large area of negative contrast due to iron disturbance. T1-weighted images assessed as showing grade 1 artefacts had hypointense local alterations caused by a strong soft tissue contrast between the normal FGT and fat. Grade 1 artefacts on T2-weighted images were located only superficially within the subcutaneous tissue and showed a clear boundary.

**Fig. 3:**
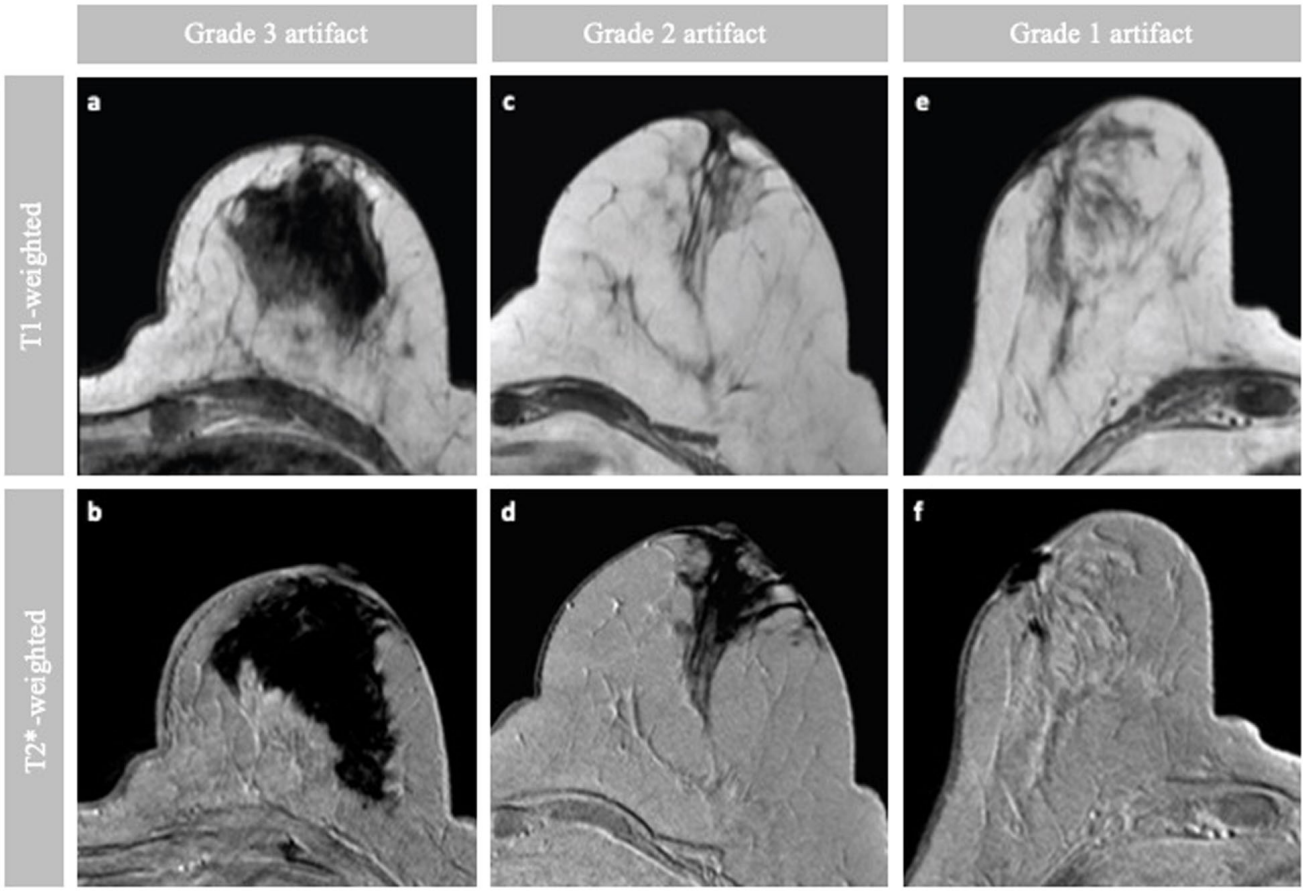
Typical axial T1-weighted and T2*-weighted images at 1.5 T. The first column (**a**, **b**) and the second column (**c**, **d**) both represent a patient in the high-dose (HD) group with grade 3 and grade 2 artefacts, respectively. The third column (**e**, **f**) represents a patient in the low-dose (LD) group with grade 1 artefacts. Grade 3 artefact: a patient from the HD group with an history of breast conserving surgery (BCS) with magnetic sentinel lymph node biopsy (SLNB) because of invasive ductal cancer (pT1c N1 M0). Follow-up imaging was performed 3 years after BCS. Grade 2 artefact: a patient from the HD group with an history of BCS with magnetic SLNB because of invasive ductal cancer (pT1 cN0 M0). Follow-up imaging was performed 3.3 years after BCS. Grade 1 artefact: a patient from the LD group with an history of BCS with magnetic SLNB because of invasive carcinoma (no special type, pT1c N0 M0). Follow-up imaging was performed 7 months after BCS.

Different severity grades of void artefacts and the time from injection to surgery grouped per severity of the artefacts is shown in Fig. 3. There were no significant differences between the HD and LD group in primary tumour size or in the volume of the excised specimen (Table 3). Table 4 summarises the results of the qualitative grading for all patients. There was a distinct difference between the two groups, with the LD group having only grade 1 artefacts and the HD group having less impaired diagnostic evaluation (grade 2 or higher) in all images. As illustrated in Table 4, a significant difference between the HD group and LD group was found in terms of percentage of patients with impaired diagnostic MRI at follow-up (*p* = 0.008, Fisher exact test).

**Table 4:** Results of qualitative grading of void artefacts for the small particle iron oxide high-dose and low-dose groups

Results of assessment	HD group *n (% of total*)	LD group *n (% of total*)
Grade 1 (follow-up MRI with good diagnostic quality)	0	6 (100%)
Grade 2 (follow-up MRI impaired but still readable)	4 (30%)	0
Grade 3 (follow-up MRI with hampered clinical assessment)	9 (70%)	0
Artefact in axillary nodes	6 (46%)	0
Artefact within the fibroglandular tissue	10 (77%)	0
Artefact on post-operative mammography	0	0

**Fig. 4:**
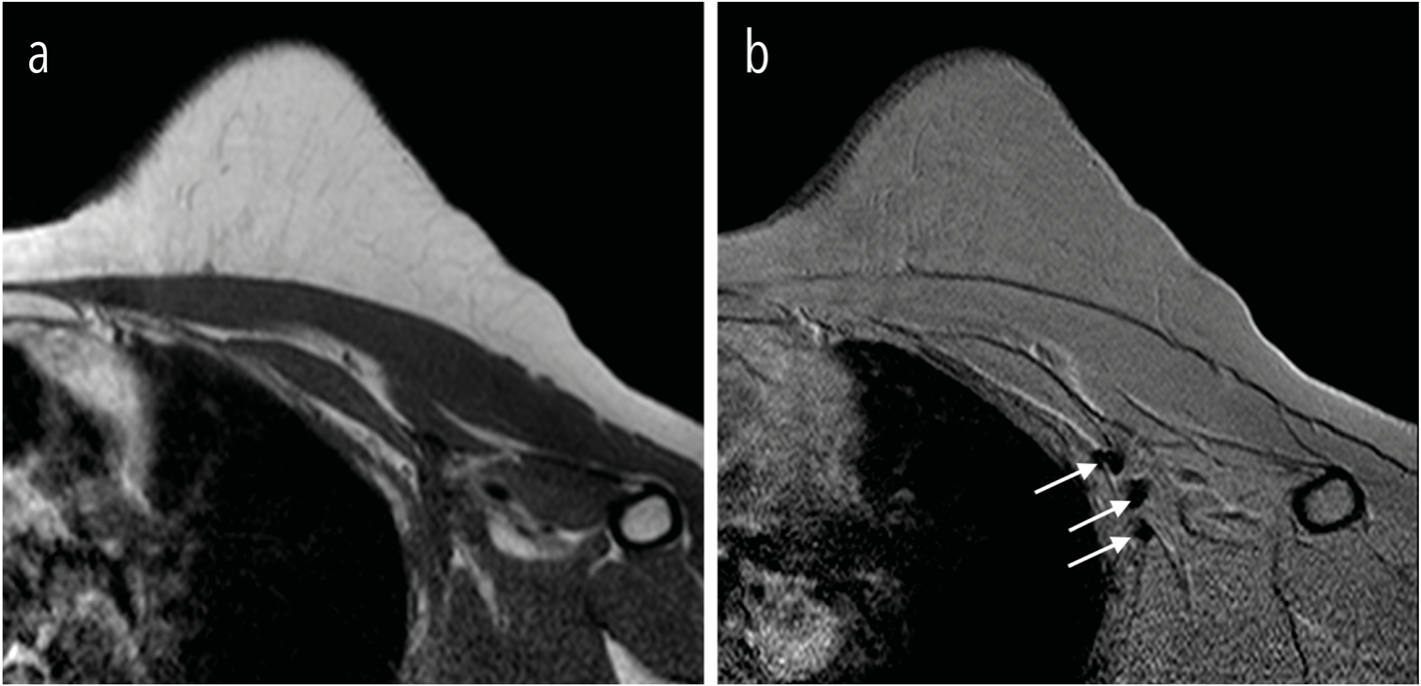
A patient from the high-dose group with an history of invasive ductal breast cancer (pT2 N2a M0). Follow-up of the axillary region was acquired 2.5 years after magnetic sentinel lymph node biopsy. **a** shows a T1-weighted image without visible small particle iron oxide (SPIO) accumulation in the lymph nodes), while **b** shows T2*-weighted image with signal voids due to SPIO accumulation in three lymph nodes

In the HD group, all 13 patients displayed a void artefact on T2*-weighted images. In 10 patients, these artefacts were located inside the FGT. The remaining three patients had a subcutaneously located artefact. On T1-weighted images, an artefact was present in 9 of 13 patients, classifying these images as unreliable by both independent observers. In the other 4 cases, image quality was impaired but still considered sufficient for diagnostic purposes. In the HD group, the residual SPIO tracer was found in the axillary region of six out of 13 patients. The axillary region of a patient from the HD group on T1-and T2-weghted images is shown Fig. 4, illustrating SPIO accumulation in the axillary lymph nodes 2.5 years after surgery. In the LD group*,* all void artefacts, observed in 3 of 6 patients, were identified only on T2*-weighted images, located superficially in the subcutaneous tissue and had a clear boundary. Considering that no artefacts were present in any of the patients on T1-weighted images, all patients in the LD group were classified as having a reliable diagnostic MRI. No SPIO artefacts were identified in the axillary region of the LD group. No SPIO residue or image disturbance was found during postoperative mammography in either the HD or LD group.

### Semiquantitative measurements

Table 5 presents the average volume of the FGT (T1-weighted images) and artefacts (T1- and T2*-weighted images) in both groups. The volume of artefact, averaged for the two observers, based upon T2*-weighted images in the HD group was 20.9 cm^3^, to be compared to 0.3 cm^3^ in the LD group. The artefact volume, averaged for the two observers, on T1-weighted images was 3.5 cm^3^ for the HD group. Both observers agreed that there was no artefact present in any of the patients on T1-weighted images in the LD group. There was no significant difference (Mann–Whitney *U* test) in absolute or relative interobserver variability (Table 5) between the HD and LD group for both volume measurements based upon T1weighted images. However, the there was a significant difference (Student-t test) in interobserver variability when assessing volume of artefact based upon T1weighted images: *i.e.*, relative interobserver variability in HD group was 8.2, compared to 0.4 in LD group.

**Table 5:** Results of quantitative grading of void artefacts volumes for high-dose and low-dose groups (cm^3^)

	Average	Absolute interobserver variability	Relative interobserver variability
HD group			
Artefact on T1-weighted images	3.5	1.9*	1.0*
Artefact on T2*-weighted images	20.9	8.2^#^	1.4^#^
Fibroglandular volume on T1-weighted images	159	55.2*	4.0*
LD group			
Artefact on T1-weighted images	None	None	None
Artefact on T2*-weighted images	0.3	0.4^#^	0.5^#^
Fibroglandular volume on T1-weighted images	213	45.4*	3.1*

*Not normally distributed

^#^Normally distributed

## Discussion

This research is a quantitative evaluation of the void artefacts due to the magnetic SLNB procedure in patients from two multicentre trials (SentiMAG [4] and MagSNOLL [12]). Subareolar administration of superparamagnetic tracer at a dose recommended by the manufacturer (2-mL tracer, subareolar injected) resulted in impaired MRI assessment up to 3 years after the SLNB. These large susceptibility artefacts could be prevented using a different injection site and dose. The administration of a LD tracer (0.1 mL) injected intratumourally prevented void SPIO-induced artefacts at follow-up, resulting into a good diagnostic quality images six months after the SLNB. The use of SPIO nanoparticles in magnetic SLNB had no influence on assessment of follow-up mammography.

There are currently only a few articles addressing the effects of SPIO nanoparticles on follow-up MRI after a magnetic SLNB procedure. Forte et al. [19], Krischer et al. [20] and Chapman et al. [24] analysed the follow-up 1.5-T or 3-T MRI of 1, 24 and, 16 patients, respectively. They all showed the impaired diagnostic value of MRI due to SPIO residue after a HD SPIO injected according to the manufacturer guidelines (2 mL into the subareolar region), which is consistent with our findings. Additionally, our study quantifies the void artefacts and their impact on MRI. Unlike a recent case report acquired under the HD protocol [25], our study did not show any effect of SPIO nanoparticles on follow-up mammography. Furthermore, our study also provides a solution to enhance diagnostic value of MRI after magnetic SLNB using an intratumoural LD SPIO injection.

The main limitations of this study involve the tracer injection in two different sites (subareolar or intratumoural) that makes it difficult to have a full and direct comparison between the two doses. However, it is expected that an intratumoural injection will provide less artefacts as this injection site is also resected. Nevertheless, the LD group clearly has the advantage of decreasing the void artefacts that might be caused by a smaller amount of residual tracer after surgery. Though imperceptible, the influence of the injection site on the perioperative detections is less prominent compared to the influence of the amount of tracer injected [14], *i.e.*, the SPIO drainage after intratumoural injection is compromised by a poor lymphatic drainage.

A further limitation is the relatively small sample size of this study, although for the HD group the results were obvious and consistent with previous studies [20, 24]. Larger artefacts were found in the HD group even though more time had elapsed between surgery and follow-up MRI (for the HD group the follow-up time was on average 3.5 years after surgery, while for the LD group it was only 1 year). This highlights that additional clearance time does not compensate for the injection site and dosage differences. Both study groups showed adequate SLN detection. In addition, a recently published work showed that a peritumoural LD SPIO injection, 1 to 7 days preoperatively, results in successful SLN detection [14]. Dynamic contrast-enhanced MRI was not included at follow-up imaging because our institute does not recommend this examination as first choice at follow-up. Literature has shown that the dynamic contrast-enhanced MRI after an iron-oxide injection is feasible in the liver [26, 27]. For breast MRI, this aspect needs to be further explored.

In conclusion, radiologists should be vigilant about the impact of SPIO nanoparticles on the follow-up MRI an impact that is potentially preventable by using a SPIO LD intratumourally injected for SLN detection. To our knowledge, this is the first study that demonstrates it is possible to safely perform a radiation free, magnetic SLNB procedure without adverse consequences at MRI follow-up.

## Data Availability

The datasets used and/or analysed during the current study are available from the corresponding author on reasonable request.

## Abbreviations

BCS: Breast conserving surgery
FGT: Fibroglandular tissue
HD: High dose
LD: Low dose
SLN: Sentinel lymph node
SLNB: Sentinel lymph node biopsy
SPIO: Superparamagnetic iron-oxide

## References

[1] registry Nc (2020) Incidentie Borstkanker Nederland 2019. Available via https://iknl.nl/kankersoorten/borstkanker/registratie/incidentie. Accessed July 16, 2020

[2] Maguire A, Brogi E (2016). Sentinel lymph nodes for breast carcinoma: an update on current practice. Histopathology.

[3] Ahmed M, Purushotham AD, Douek M (2014). Novel techniques for sentinel lymph node biopsy in breast cancer: a systematic review. Lancet Oncol.

[4] Douek M, Klaase J, Monypenny I (2014). Sentinel node biopsy using a magnetic tracer versus standard technique: the SentiMAG Multicentre Trial. Ann Surg Oncol.

[5] Ghilli M, Carretta E, Di Filippo F (2017). The superparamagnetic iron oxide tracer: a valid alternative in sentinel node biopsy for breast cancer treatment. Eur J Cancer Care (Engl).

[6] Houpeau JL, Chauvet MP, Guillemin F, et al (2016) Sentinel lymph node identification using superparamagnetic iron oxide particles versus radioisotope: the French Sentimag feasibility trial. J Surg Oncol 113:501–507. 10.1002/jso.24164 10.1002/jso.24164 26754343

[7] Karakatsanis A, Christiansen PM, Fischer L, et al (2016) The Nordic SentiMag trial: a comparison of super paramagnetic iron oxide (SPIO) nanoparticles versus Tc(99) and patent blue in the detection of sentinel node (SN) in patients with breast cancer and a meta-analysis of earlier studies. Breast Cancer Res Treat 157:281–294. 10.1007/s10549-016-3809-9 10.1007/s10549-016-3809-9 PMC4875068 27117158

[8] Pinero-Madrona A, Torro-Richart JA, de Leon-Carrillo JM (2015). Superparamagnetic iron oxide as a tracer for sentinel node biopsy in breast cancer: a comparative non-inferiority study. Eur J Surg Oncol.

[9] Rubio IT, Diaz-Botero S, Esgueva A, et al (2015) The superparamagnetic iron oxide is equivalent to the Tc99 radiotracer method for identifying the sentinel lymph node in breast cancer. Eur J Surg Oncol 41:46–51. 10.1016/j.ejso.2014.11.006 10.1016/j.ejso.2014.11.006 25466980

[10] Thill M, Kurylcio A, Welter R, et al (2014) The Central-European SentiMag study: sentinel lymph node biopsy with superparamagnetic iron oxide (SPIO) vs. radioisotope. Breast 23:175–179. 10.1016/j.breast.2014.01.004 10.1016/j.breast.2014.01.004 24484967

[11] Zada A, Peek MC, Ahmed M (2016). Meta-analysis of sentinel lymph node biopsy in breast cancer using the magnetic technique. Br J Surg.

[12] Ahmed M, Anninga B, Goyal S, et al (2015) Magnetic sentinel node and occult lesion localization in breast cancer (MagSNOLL Trial). Br J Surg 102:646–652. 10.1002/bjs.9800 10.1002/bjs.9800 25868072

[13] Warnberg F, Stigberg E, Obondo C (2019). Long-term outcome after retro-areolar versus peri-tumoral injection of superparamagnetic iron oxide nanoparticles (SPIO) for sentinel lymph node detection in breast cancer surgery. Ann Surg Oncol.

[14] Karakatsanis A, Pistioli L, Bagge RO, et al (2020) SentiDose Trial: Optimizing dose and injection timing in magnetic sentinel node detection for early breast cancer. European Journal of Cancer 138:S45–S45. 10.1016/S0959-8049(20)30644-4

[15] Houssami N, Turner RM, Morrow M (2017). Meta-analysis of pre-operative magnetic resonance imaging (MRI) and surgical treatment for breast cancer. Breast Cancer Res Treat.

[16] Lam DL, Houssami N, Lee JM (2017). Imaging surveillance after primary breast cancer treatment. AJR Am J Roentgenol.

[17] Bakker MF, de Lange SV, Pijnappel RM, Mann RM, et al (2019) Supplemental MRI screening for women with extremely dense breast tissue. N Engl J Med 381:2091–2102. 10.1056/NEJMoa1903986 10.1056/NEJMoa1903986 31774954

[18] Shah C, Ahlawat S, Khan A, Tendulkar RD, Wazer DE, Shah SS, Vicini F (2016). The role of MRI in the follow-up of women undergoing breast-conserving therapy. Am J Clin Oncol.

[19] Forte S, Kubik-Huch RA, Leo C (2019). Improvement in breast magnetic resonance imaging after a sentinel procedure for breast cancer with superparamagnetic tracers. Eur J Radiol Open.

[20] Krischer B, Forte S, Niemann T, Kubik-Huch RA, Leo C (2018). Feasibility of breast MRI after sentinel procedure for breast cancer with superparamagnetic tracers. Eur J Surg Oncol.

[21] Ltd E (2012) Productinformation Sienna+® en SentiMag®. In: Ltd E, (ed) CE 563405, Cambridge

[22] Huizing E, Anninga B, Young P, Monypenny I, Hall-Craggs M, Douek M (2015). Analysis of void artefacts in post-operative breast MRI due to residual SPIO after magnetic SLNB in SentiMAG Trial participants. Europ J Surg Oncol.

[23] Christenhusz A, Pouw J, Alic L (2020). Assessing void artifacts in follow-up breast MRI scans after sentinel node biopsy using superparamagnetic tracer.

[24] Chapman MC, Lee AY, Hayward JH, Joe BN, Price ER (2020). Superparamagnetic iron oxide sentinel node tracer injection: effects on breast MRI quality. J Breast Imaging.

[25] Arslan G, Yilmaz C, Celik L, Cubuk R, Tasali N (2019). Unexpected finding on mammography and MRI due to accumulation of iron oxide particles used for sentinel lymph node detection. Europ J Breast Health.

[26] Kubaska S, Sahani DV, Saini S, Hahn PF, Halpern E (2001). Dual contrast enhanced magnetic resonance imaging of the liver with superparamagnetic iron oxide followed by gadolinium for lesion detection and characterization. Clin Radiol.

[27] Semelka RC, Lee JK, Worawattanakul S, Noone TC, Patt RH, Ascher SM (1998). Sequential use of ferumoxide particles and gadolinium chelate for the evaluation of focal liver lesions on MRI. J Magn Reson Imaging.

